# Factors associated with utilization of motorcycle ambulances by pregnant women in rural eastern Uganda: a cross-sectional study

**DOI:** 10.1186/s12884-016-0808-0

**Published:** 2016-03-03

**Authors:** Rogers Ssebunya, Joseph K. B. Matovu

**Affiliations:** Makerere University College of Health Sciences, School of Public Health, P.O. Box 7072, Kampala, Uganda

**Keywords:** Motorcycle ambulance, pregnant women, health facility deliveries

## Abstract

**Background:**

Evidence suggests that use of motorcycle ambulances can help to improve health facility deliveries; however, few studies have explored the motivators for and barriers to their usage. We explored the factors associated with utilization of motorcycle ambulances by pregnant women in eastern Uganda.

**Methods:**

This was a cross-sectional, mixed-methods study conducted among 391 women who delivered at four health facilities supplied with motorcycle ambulances in Mbale district, eastern Uganda, between April and May 2014. Quantitative data were collected on socio-demographic and economic characteristics, pregnancy and delivery history, and community and health facility factors associated with utilization of motorcycle ambulances using semi-structured questionnaires. Qualitative data were collected on the knowledge and attitudes towards using motorcycle ambulances by pregnant women through six focus group discussions. Using STATA v.12, we computed the characteristics of women using motorcycle ambulances and used a logistic regression model to assess the correlates of utilization of motorcycle ambulances. Qualitative data were analyzed manually using a master sheet analysis tool.

**Results:**

Of the 391 women, 189 (48.3 %) reported that they had ever utilized motorcycle ambulances. Of these, 94.7 % were currently married or living together with a partner while 50.8 % earned less than 50,000 Uganda shillings (US $20) per month. Factors independently associated with use of motorcycle ambulances were: older age of the mother (≥35 years vs ≤24 years; adjusted Odds Ratio (aOR) = 4.3, 95 % CI: 2.03, 9.13), sharing a birth plan with the husband (aOR = 2.5, 95 % CI: 1.19, 5.26), husband participating in the decision to use the ambulance (aOR =3.22, 95 % CI: 1.92, 5.38), and having discussed the use of the ambulance with a traditional birth attendant (TBA) before using it (aOR =3.12, 95 % CI: 1.88, 5.19). Qualitative findings indicated that community members were aware of what motorcycle ambulances were meant for and appreciated their role in taking pregnant women to health facilities.

**Conclusion:**

The use of motorcycle ambulances was associated with older age of the mother, male participation in birth preparedness, and consultations with TBAs. These findings suggest a need for interventions to involve men in reproductive health as well as efforts to reach women younger than 35 years of age.

**Electronic supplementary material:**

The online version of this article (doi:10.1186/s12884-016-0808-0) contains supplementary material, which is available to authorized users.

## Background

Evidence shows that maternal mortality rates can be substantially reduced if all mothers were able to deliver at a health facility [1]. However, although the proportion of women who delivered at the hands of skilled personnel has increased from 59 % in 1990 to 70 % in 2013 globally, in sub-Saharan Africa, this proportion remains below 50 % [2, 3]. Studies show that many pregnant women, especially in sub-Saharan Africa, continue to deliver at the hands of traditional birth attendants or at home [4–6]. Nearly one in four mothers deliver alone at home or with the assistance of a relative [7], creating a missed opportunity for skilled management of the delivery process. The consequences arising from lack of skilled birth attendance are usually fatal [8–10], with both maternal and neonatal mortality increasing among women delivering outside formal health care systems. In Uganda, only 57 %[11] of the women deliver in health facilities suggesting that a high proportion of women either deliver at home or along the way to health facilities or at traditional birth attendants’ (TBA) homes. As a result, maternal mortality remains high at 438/100,000 live births [11], partly because most women don’t have access to a skilled birth attendant.

Several barriers have been documented to explain the lack of skilled attendance among pregnant women in sub-Saharan Africa including lack of professional care providers [7] poor quality of maternity services due to inadequate knowledge and skills among health care providers [12, 13], lack of privacy, lack of means of transport [5] and absence of decision making power [5, 7]. Recent studies show that some of these barriers can be minimized through use of alternative means of transport including; the use of a locally made *Ingobyi* in Rwanda [14], stretchers in Nepal [15], use of bicycles in Malawi, Zambia and Uganda [16–18] and use of motorcycle ambulances in Malawi, Sierra Leone, South Sudan and Uganda [19–23]. Improving access to maternal health services with alternative transport means has been identified as a prominent pillar in improving maternal and child health indicators especially in developing nations. Evidence from published studies and project reports has illustrated this vital role. The World Health organization [24], UKaid [25], and AusAID [26] have all identified and acknowledged prominently the need for innovations in improving access to health facilities by pregnant women, including use of motorcycle ambulances [24].

Motorcycle ambulances work by transporting patients, especially pregnant women, from periphery health facilities or rural communities to district hospitals and the nearby health units that can offer emergency obstetric care and are operated by trained drivers [19, 20, 24]. This means that motorcycle ambulances can play a crucial role in reducing maternal and neonatal mortality by helping women to deliver at health facilities. A study in Malawi found that the use of motorcycle ambulances increased health facility-based maternal deliveries from 25 to 49 % and reduced the occurrence of maternal mortality from 586 to 235 per 100,000 live births in three years [24]. This was because these ambulances were stationed near the periphery health units and women could easily access them. However, while a lot of attention has been paid to how motorcycle ambulances work and their role in improving maternal and child health, most studies have focused on the costs of operations and referral time [19], the perceptions towards their utilization [21], conditions of patients transported using the ambulances and the accessibility and feasibility of using them [20]. There is limited documentation on the factors affecting the utilization of motorcycle ambulance among pregnant women living within the catchment area of health facilities served by these ambulances. In this study, we contribute to the existing literature on motorcycle ambulances by exploring these factors in order to inform the future promotion and eventual utilization of this essential means of transport.

## Methods

### Study design, population and site

This was a cross-sectional, mixed-methods study that was conducted in Mbale district, eastern Uganda, using quantitative and qualitative data collection methods. The study was carried out among women who delivered in four health facilities that had motorcycle ambulances in 2013. The district has a population of 492,804 (2014 census estimates) with about 99,546 women in child bearing age. Mbale is a rural district with majority of the people living in the villages. It has 61 health facilities including one public regional referral hospital, two private hospitals, four health centers (HC) IVs, 23 HC IIIs and 34 HC IIs. Each health center IV serves as a referral center for obstetric cases from the lower-level health centers and the communities.

### Introduction and implementation of the motorcycle ambulances referral system

Because of its hilly nature, transport from the rural parts of the district is difficult especially during the rainy season. Many people in the community are peasants with limited ability to afford, let alone, use private means of transport. In addition, cultural beliefs and the value attached to the placenta by women after birth [27] continue to hamper women’s willingness to deliver at health facilities. As a result, fewer women deliver at health facilities, with many opting to deliver at home or at the traditional birth attendants’ place. In view of these challenges, Mbale district introduced the use of motorcycle ambulances with the objective of increasing health facility deliveries which would in return help to reduce the high maternal mortality ratio, estimated at 567 per 100,000 live births in Mbale district (J Waniaye, personal communication).

In 2010, Mbale district partnered with a local non-governmental organization; Partnership Overseas Networking Trust (PONT), to provide four motorcycle ambulances to four health facilities, i.e., Wanale HC III, Makhonje III, Busiu HC IV, and Namanyonyi HC III. Each health facility was assigned one well trained driver to man the ambulance. All ambulance services were provided free of charge; riders were provided with a monthly stipend and airtime for communication purposes from the district. About 30 traditional birth referral attendants (TBRA) were trained to work with the ambulance drivers so that pregnant women identified as needing emergency obstetric care could be referred to any of the participating health facilities using the ambulances. It was the responsibility of the TBRA in each village to call the facility where the ambulance was stationed and/or the ambulance drivers whenever there was need for referral. Communities were sensitized about the importance of motorcycle ambulances through community outreaches. TBRAs were provided with cell phones and their contacts were made known to village health team members to contact the drivers whenever a referral was necessary. Ambulance drivers didn’t have skills to conduct a delivery but TBRAs were trained to identify mothers with complications, call for an ambulance and escort the mother to the health facility while in transit on a motorcycle ambulance as needed (F Chemuto, personal communication).

### Sample size determination and sampling procedures

We obtained a representative number of women from each facility through stratified sampling procedures using a probability proportional to size strategy. A sample size of 391 women was estimated using Kish Leslie (1965) formula, assuming prevalence of use of the motorcycle ambulances at 20 %, a precision of 5 %, a standard normal deviation at 1.96 and an adjustment for non-response of 2 % [28]. Using maternity registers at each health facility, we obtained information (names, address, and telephone contact where available) on all women who delivered at each facility between January and December 2013, and generated numbered lists of all eligible women. Women were eligible to participate in the study if they delivered at any of the participating health facilities between January and December 2013; lived within the catchment area of the health facility supplied with a motorcycle ambulance; and were recorded in the maternity register. Using an online table of random numbers generator [29], specific numbers were selected and the names of women corresponding to these numbers were selected to participate in this study. This was done for each of the participating health facilities until the sample size was obtained. Women below 18 years included in this study were emancipated minors who were able to provide informed consent as guided by the national guidelines [30].

### Selection of qualitative interview participants

Qualitative interview participants were both men and women living in the catchment area of the health facilities supplied with motorcycle ambulances. They were all selected purposively. Women were selected to participate in the study if they had delivered in the previous two years at the participating health facilities but used other means of transport to access delivery services. On the other hand, men were selected to participate in the study as long as they had wives who were still in the reproductive age group (15–49 years). Participants were identified with the assistance of village health team (VHT) Chairpersons and the Community Development Officer (CDO). To facilitate the identification process, we informed the VHT Chairperson or the CDO about the enrolment criteria (as described above), and whenever we approved a household, either the VHT chairperson or CDO helped the research team to identify and verify the individuals that were eventually invited for the qualitative interviews.

### Data collection procedures

Quantitative data were collected between April and May 2014. Interviewer-administered, semi-structured questionnaires (See Additional File 1) were administered to consenting women who delivered at each of the four participating health facilities between January and December 2013. Data were collected on respondents’ characteristics, community factors, and health service factors. Respondents’ characteristics included; socio-demographic (age of the respondents, marital status, occupation, tribe, religion, level of education) and economic (monthly income, ownership of a phone) characteristics, pregnancy and delivery history (number of antenatal visits attended during that pregnancy, history of any obstetric complication) and motorcycle ambulance use-related characteristics. Community factors included; women not wanting to be seen in public while in labour and the cultural expectation that pregnant women should not sit on a motorcycle ambulance. Health service factors included; availability of obstetric drugs and supplies for delivery in the health facility, whether or not women believed that ambulance drivers have obstetric skills, health workers’ and drivers’ attitudes towards pregnant women in labour, whether or not women discussed with traditional birth attendants on whether to use or not to use a motorcycle ambulance during the course of her previous pregnancy and distance of the women’s home from the health facilities.

Data were collected by four trained research assistants fluent in the local languages (*Lumasaba* and *Luganda*) and English. Women were traced and interviewed at home. At least two repeat visits were made to locate and interview all the eligible women. Those who were not found at home at the second attempt to locate were replaced with other women randomly selected from the maternity register that was used to select the initial cohort of women. All completed questionnaires were edited in the field to ensure completeness and accuracy; returned to a central office location on a daily basis and entered into Epi-data version 3.02.

Qualitative data were collected using focus group discussion (FGD) guides (See Additional File 2). Data were collected on level of awareness, attitudes towards the ambulances, barriers and facilitators to using the ambulances, and suggestions for improvement on the functionality of the ambulances by a team of research assistants comprising a moderator and a note-taker. Interviews were conducted at the health facilities where the ambulances are stationed, and lasted for a period of one and a half hours on average. All data were audio-recorded with permission from the study participants. Six FGDs (3 for males and 3 for females) were conducted among 28 women and 29 men; with each FGD comprising of 8–10 participants. Male discussants were within the age group of 20–50 while females were within 20–45 years. Men and females were separated in these discussions to allow freedom of speech from all participants. FDGs were conducted in *Luganda*/*Lumasaba*/English concurrently for better understanding and all participants consented before the discussions.

### Measures

The primary outcome, utilization of motorcycle ambulances, was defined as a binary variable with Yes = 1 indicating that a pregnant woman used the ambulance to go to the health facility at the time of delivery and No = 2 indicating that a pregnant woman used other forms of transport to go to the facility. In this study, the term married was used to mean all forms of marriage; married in church/mosque, traditionally married or civil marriage. The term “living together” was used to mean all women who might have not been married in any of the above described forms of marriage but were living together with their spouses. The variable “occupation” was categorized into peasant, student, civil servant, business woman and others. We used the term “peasant” to refer to a woman who was engaged in subsistence farming, with no expected monthly salary. Women who said they earn some money during a typical month were asked to state how much money they earn per month, and the figures presented are based on self-reports.

### Data analysis

At the time of analysis, all quantitative data were transferred from EpiData to STATA statistical software version 12. We computed descriptive statistics to obtain characteristics of the study respondents. Categorical variables were expressed using frequencies and percentages while continuous variables were expressed as mean (standard deviation). A Chi Square test was used to assess for differences in proportions among women who used and those who didn’t use the ambulances. A logistic regression model was used to identify factors that were statistically associated with the primary outcome both at the bivariate and multivariable levels. All factors at the bivariate level that had a *p*-value of less than 0.2 were selected for inclusion into the multivariable logistic regression model. A *p*-value less than 0.05 was considered significant at the multivariable analysis level.

Qualitative data were transcribed verbatim and entered into a Microsoft Word processing document for analysis. Data were analyzed manually using a master sheet analysis tool. All transcripts were printed out and read through by the first author to identify emerging issues based on a priori themes. These themes included knowledge about the existence of the motorcycle ambulances, attitudes towards use of motorcycle ambulances, barriers and facilitators to usage, and suggestions for improvement their role. Relevant quotations were identified and used to support each theme during the reporting process.

### Ethical considerations

This study was approved by the Institutional Review Board (IRB) of Makerere University School of Public Health (MakSPH) before conducting the study. Permission was also obtained from the district health officer (Mbale). Written informed consent was obtained from the respondents participating in the study. Data collection tools were coded and no names of respondents appeared anywhere for confidential purposes. The study was adherent to the STROBE criteria as outlined in Additional file 3.

## Results

### Quantitative findings

#### Population characteristics

Table 1 shows the characteristics of the 391 women who participated in the study. Of these, 187 (47.8 %) were within the age of 15–24 years while 147 (37.6 %) were aged 25–34 years. Majority (71.1 %) of the women had primary education, 362 (92.6 %) were currently married/ living together while 208 (53.2 %) earned less than 50,000 Uganda shillings (US $ 20) per month. Among the 391 women interviewed, 189 (48.3 %) reported current or most recent use of the ambulances while 202 (51.7 %) didn’t. Of those who used the ambulances (189), 90 % were peasants, while 50.8 % earned less than 50,000 Uganda shillings (US$ 20) per month (Table 1).

**Table 1:** Characteristics of the study population

Variable	*n* = 391	Percent
Age group		
15–24	187	47.8
25–34	147	37.6
35+	57	14.6
Education level		
≤ Primary Level	278	71.1
≥ Secondary Level	113	28.9
Marital status		
Currently married/living together	362	92.6
Other^b^	29	7.4
Religion		
Anglican	170	43.5
Catholics	75	19.2
Muslims	108	27.6
Other Christians^a^	38	9.7
Tribe		
Bagisu	322	82.4
Other tribe	69	17.7
Occupation of the woman		
Peasant	339	86.7
Business woman	25	6.4
Other occupation	27	6.9
Estimated monthly income		
<50,000	208	53.2
≥50,000	183	46.8
Use of mA		
Yes	189	48.3
No	202	51.7
Health facility level		
Health Center IV	258	66.0
Health Center III	133	34.0

^a^ − Pentecostals and Seventh Day Adventists, ^b^ − Never married, Divorced/separated, widowed

Compared to those who did not use motorcycle ambulances, those who used the ambulances were more likely to; belong to the Bagisu tribe (Bagisu: 88.9 % vs 76.2 %, *p* = 0.001) and to be Catholics (24.3 % vs 14.4 %; *p* = 0.04) . Also, motorcycle ambulance users were more likely to be older (e.g. 25–35 years: 41.8 % vs 33.7 %, *p* = 0.001) than non-users. There was no significant difference in the proportions of users and non-users regarding level of education, marital status, occupation and average monthly income (Table 2).

**Table 2:** Characteristics of motorcycle ambulance users and non-users by socio-demographic characteristics

	Used mA		
	Yes (*N* = 189)	No (*N* = 202)	*P*-value
Variable	n (%)	n (%)	
Age group			0.001*
15–24	67 (35.5)	120 (59.4)	
25–34	79 (41.8)	68 (33.7)	
35+	43 (22.8)	14 (6.9)	
Mean Age(SD)	26.4 (6.9)	26.3 (6.82)	
Religion			0.039*
Anglican	77 (40.7)	93 (46.0)	
Catholics	46 (24.3)	29 (14.4)	
Muslims	45 (23.8)	63 (31.2)	
Other Christians	21 (11.1)	17 (8.4)	
Tribe			
Bagisu	168 (88.9)	154 (76.2)	0.001*
Other tribe	21 (11.1)	48 (23.8)	
Education level			0.890
≤ Primary Level	135 (71.4)	143 (70.8)	
≥ Secondary Level	54 (28.6)	59 (29.2)	
Occupation			0.167
Peasant	170 (90.0)	169 (83.7)	
Business woman	10 (5.3)	15 (7.4)	
Other occupation	9 (4.8)	18 (8.9)	
Marital status			0.121**
Currently married/ Living together	179 (94.7)	183 (90.6)	
Others^a^	10 (5.29)	19 (9.4)	
Monthly Income			0.357
<50,000	96 (50.8)	112 (55.5)	
≥50,000	93 (49.2)	90 (44.55)	

**significant at *p* < 0.2, *significant at *p* < 0.05, ^a^Never married, divorced, separated or widowed

#### Predictors of the use of motorcycle ambulances

Table 3 shows the association between use of motorcycle ambulances and selected socio-demographic characteristics at the bivariate analysis level. Results show that women aged 25–34 (OR = 2.1, 95 %CI; 1.34, 3.23) and those aged 35 or more years (35+ years) (OR = 5.5, 95 %CI; 2.81, 10.78) were more likely to use a motorcycle ambulance during delivery than those aged 15–24 years. Women who shared a birth plan with their husbands (OR = 3.65, 95 %CI; 2.0, 6.67) and those whose husbands participated in the decision to use a motorcycle ambulance (OR = 3.78, 95 %CI; 2.46, 5.81) were nearly four times more likely to use a motorcycle ambulance than those who didn’t. Other factors that were significantly associated with utilization of motorcycle ambulances at the bivariate analysis level included discussing with a traditional birth attendant before going to the health facility to deliver (OR = 3.6, 95 %CI; 2.3, 5.65), having attended more than four ANC visits during pregnancy (OR = 1.93, 95 % CI, 1.20, 3.08), living far away from the health facilities (>5kms) that were allocated the ambulances (OR = 1.75, 95 % CI; 1.14, 2.69), disagreeing that drivers had poor attitudes towards pregnant women in labour (OR = 0.22, 95 % CI; 0.09, 0.56) and believing that the ambulance drivers had some obstetric skills (OR = 2.21, 95 % CI; 1.23, 3.97).

**Table 3:** Association between utilization of motorcycle ambulances by pregnant women and socio-demographic characteristics

	Used mA				
Variable	Yes n (%)	No n (%)	Unadjusted OR	95 % CI	*p*
Age group					
15–24	67 (35.5)	120 (59.4)	1.0 (ref)		
25–34	79 (41.8)	68 (33.7)	2.1	1.34–3.23	0.001*
35+	43 (22.8)	14 (6.9)	5.50	2.81–10.78	0.001*
Religion					
Anglican	77 (40.7)	93 (46.0)	1.0 (ref)		
Catholic	46 (24.3)	29 (14.4)	1.92	1.10–3.33	0.021*
Muslims	45 (23.8)	63 (31.2)	0.86	0.53–1.40	0.553
Other Christians	21 (11.1)	17 (8.4)	1.49	0.74–3.03	0.267
Tribe					
Other Tribe	21 (11.1)	48 (23.8)	1.0 (ref)		
Bagisu	168 (88.9)	154 (76.2)	2.49	1.43–4.35	0.001*
Marital status					
Others	10 (5.29)	19 (9.4)	1.0 (ref)		
Married, Living together	179 (94.7)	183 (90.6)	1.86	0.84–4.11	0.126**
Sharing birth plan with husband					
No	16 (8.5)	51 (25.3)	1.0 (ref)		
Yes	173 (91.5)	151 (74.8)	3.65	2.0–6.67	0.001*
Husband participation in using mA					
No	49 (25.9)	115 (56.9)	1.0 (ref)		
Yes	140 (74.1)	87 (43.1)	3.78	2.46–5.80	0.001*
Would consult HW first before using mA					
No	149 (78.8)	121 (59.9)	1.0 (ref)		
Yes	40 (21.2)	81 (40.1)	0.40	0.26–0.63	0.001*
Consulted TBA before using mA					
No	175 (92.6)	171 (84.7)	1.0 (ref)		
Yes	14 (7.4)	31 (15.4)	0.44	0.23–0.86	0.016*
mA tends to delay					
Disagree	144 (76.2)	137 (67.8)	1.0 (ref)		
Agree	45 (23.8)	65 (32.2)	0.66	0.42–1.02	0.067**
Culture allows a pregnant woman to sit on a motorcycle					
No	36 (19.1)	27 (13.4)	1.0 (ref)		
Yes	153 (90.0)	175 (86.6)	0.66	0.38–1.13	0.127**
ANC visit					
≤3 visits	36 (19.1)	63 (31.2)	1.0 (ref)		
≥4 visits	153 (81.0)	139 (68.8)	1.93	1.20–3.08	0.006*
Presence of obstetric drugs/ supplies in health facility					
No	45 (23.8)	30 (14.9)	1.0 (ref)		
Yes	144 (76.2)	172 (85.2)	0.56	0.33–0.93	0.025*
Prefer mA to be escorted with midwife					
Disagree	13 (6.9)	34 (16.8)	1.0 (ref)		
Agree	176 (93.1)	168 (83.2)	2.74	1.40–5.37	0.003*
Discussed with TBA first before transit					
No	100 (52.9)	162 (80.2)	1.0 (ref)		
Yes	89 (47.1)	40 (19.8)	3.60	2.30–5.65	0.001*
Opinion that drivers’ attitudes are very poor					
Agree	166 (87.8)	196 (97.0)	1.0 (ref)		
Disagree	23 (12.2)	6 (3.0)	0.22	0.09–0.56	0.001*
Living far away from HF with mA					
Disagree	50 (26.5)	78 (38.6)	1.0 (ref)		
Agree	139 (73.5)	124 (61.4)	1.75	1.14–2.69	0.011*
Opinion that mA drivers have some obstetric skills					
No	19 (10.1)	40 (19.8)	1.0 (ref)		
Yes	170 (90)	162 (80.2)	2.21	1.23–3.97	0.008*

** significant at *p* < 0.2 , *significant at *p* < 0.05

Table 4 shows the adjusted odds ratios and 95 % confidence intervals associated with use of a motorcycle ambulance among pregnant women in Mbale district. As shown, older women (i.e. ≥35 years of age) were more likely to use a motorcycle ambulance than those ≤ 24 years of age (adjusted OR (aOR) = 4.3, 95 % CI: 2.03, 9.13). Also, pregnant women who shared their birth plans with their husbands (aOR = 2.5, 95 % CI: 1.19, 5.26) and pregnant women whose husbands participated in the decision to use a motorcycle ambulance during labour (aOR =3.22, 95 % CI: 1.92, 5.38) were significantly more likely to use motorcycle ambulances than their counterparts. Similarly, women who discussed their intention to use a motorcycle ambulance with a traditional birth attendant (TBA) during their previous pregnancies (aOR =3.12, 95 % CI: 1.88, 5.19) and those who preferred a midwife to escort them while in transit (aOR = 3.1, 95 % CI: 1.41, 6.79) were also significantly more likely to use motorcycle ambulances than their counterparts.

**Table 4:** Crude and adjusted Odds ratios of utilization of motorcycle ambulance and covariates

Variable		Number	Percent	UOR	95 % CI	aOR	95 % CI	*P*
Age group	15–24 (ref)	67	35.5	1.0				
	25–34	79	41.8	2.1	1.34–3.23	1.84	1.12–3.04	0.017*
	35**+**	43	22.8	5.5	2.81–10.78	4.30	2.03–9.13	0.001*
Sharing a birth plan with husband	No (ref)	16	8.5					
	Yes	173	91.5	3.7	2.0–6.67	2.5	1.19–5.26	0.016*
Husband’s participation in a woman’s decision to use mA	No (ref)	49	25.9					
	Yes	140	74.1	3.8	2.46–5.80	3.22	1.92–5.38	0.001*
Pregnant women preferring a midwife to escort mA	No (ref)	13	6.9					
	Yes	176	93.1	2.7	1.40–5.37	3.1	1.41–6.79	0.005*
Discussed with TBA first on how to come to HF	No (ref)	100	52.9					
	Yes	89	47.1	3.6	2.30–5.65	3.12	1.88–5.19	0.001*
Women’s opinion that driver’s attitudes are poor	Agree (ref)	166	87.8					
	Disagree	23	12.2	0.22	0.09–0.56	0.23	0.08–0.70	0.009*
Women’s opinion that drivers have some obstetric skills^a^	No (ref)	19	10.1					
	Yes	170	90.0	2.2	1.23–3.97	1.93	0.97–3.85	0.063

*UOR* Unadjusted Odds Ratio, *HF* Health facility, *TBA* Traditional Birth Attendant

^a^Drivers’ skills here meant the ability to provide any emergence help while woman is in labour like conducting the delivery, * *P* < 0.05 and Significant at 95 % confidence interval

### Qualitative findings

Qualitative findings have been categorized by theme into: knowledge about motorcycle ambulances, attitudes towards their use, facilitators and barriers, and suggestions for improving service delivery, as indicated in the following sub-sections.

#### Knowledge about the motorcycle ambulance use

The majority of the participants in the six FGDs were aware of the existence of the motorcycle ambulances and what they were meant to do:

“*Yes, I have heard about it [the motorcycle ambulance] and even seen it. It is a very useful mode of transport in the village here. It has even carried my pregnant daughter to the health center when she went into labour and we had no other means of transport*” (FGD, women, Lwangasa village, Wanale sub county)

However, some participants were still not knowledgeable about which kinds of patients were meant to be carried by the ambulances as expressed in the following statement:

“*These ambulances… I know that [they are] supposed to help us poor people in the village. When our wives get problems, when they are heavy we call the piki [i.e. motorcycle ambulance], when our babies become very sick, we call the piki. But even me, if I get a serious problem, I can call the piki*” (FGD, men, Makhonje village, Busui Sub-County)

#### Attitudes towards motorcycle ambulance use

The use of motorcycle ambulances to transport pregnant women to health facilities is highly appreciated by most members of the community. Participants in all FDGs were generally appreciative of the government’s offer of these ambulances as indicated in the quotation below:

“*For me I can talk only good things … in February my wife started to feel pains for giving birth at 4am. I called the driver and he came to my help. My wife came to hospital and gave me a very beautiful boy. If it was not like that I would have used a lot of money or my wife would have produced at home. Who would have helped her?*” (FGD, women, Makhonje village, Busui Sub-County)

#### Facilitators of and barriers to using motorcycle ambulances

We asked participants about what facilitates and hinder pregnant women from using the motorcycle ambulances. The main facilitators generally rotated around drivers being good to the pregnant women, the ambulance service being provided free of charge, and availability of the ambulance as can be seen from the quotations below;

*“You see our driver is a very good man, he comes when you need him. He operates in many health centers. Can you imagine even when you call the ambulance at it will carry you free of charge…”* (FGD, men, Busiu Township, Busiu Sub-county)

*“…at least the driver is good and he does not ask for money. The man talks very well even when he is very tired”* (FGD, women, Makhonje village, Busui Sub-county)

On the issue of barriers to use, participants’ views generally reflected financial and accessibility-related constraints. The quotations below show what participants reported in as far as these barriers are concerned;

*“…for me who stays a bit away from the road and in the hills far up, it does not help me very much because there is no road for it to pass. We carry our patients on the backs until we reach the roadside then the ambulance can carry the patient” (*FGD, women, Lwangasa village, Wanale sub county)

“*My friends, I know the piki [i.e. motorcycle ambulance] is a good thing but there are some problems we get. You see Busiu is a very big place and one ambulance is not enough and another thing is that sometimes the patients need the ambulance but they find it has no fuel…”* (FGD, men, Makhonje village, Busiu Sub County)

#### Suggestions for improvements on the use of motorcycle ambulances

Participants were asked to provide suggestions on what needs to be done to improve the use of motorcycle ambulances by pregnant women. Suggestions were generally aimed at eliciting ways of enhancing the utilization of the ambulances, as shown in the quotations below:

*“…So I think they should get a reserve driver so that when he [the current motorcycle ambulance driver] gets a problem, the other one can help”* (FGD, men, Makhonje village, Busiu Sub County)

*“…for me I mentioned a challenge but my friends didn’t understand me. The chairman said, that sometimes when there is no fuel, we should learn to have sometime 5,000/= to put fuel to save our patient”* (FGD, men, Busiu township, Busiu Sub-county)

## Discussion

Our study of the factors affecting utilization of motorcycle ambulances by pregnant women in Mbale district, Uganda, found that utilization of motorcycle ambulances is associated with older age of the mother (aged 35+ years), involvement of the male partner in the discussion of whether or not to use a motorcycle ambulance, women sharing birth plans with their husbands, and women consulting traditional birth attendants before using the motorcycle ambulances. These findings are consistent with those from other studies [31–34] and suggest that interventions aimed at improving utilization of motorcycle ambulances should focus on reaching younger women (i.e. below 35 years) and husbands of women in reproductive age as well as involving traditional birth attendants in birth preparedness.

The finding that women who used the ambulances were more likely to be aged 35 years or more suggests that many of the young mothers (<35 years) either did not deliver at the health facilities or if they did, they used other means of transport. Studies in Brazil [32], South Africa [33] and India [35] have reported similar findings, suggesting that older women tend to utilize emergence obstetric care much more than young women. It is likely that older women are more confident and less ashamed to use motorcycle ambulances than younger women who may have preferred to use other means of transport. However, this is just a possibility, and may not fully explain the observed finding since we did not collect data on what other means of transport that pregnant women used to reach the health facilities.

We found that women who involved their husbands in birth planning were more likely to utilize motorcycle ambulances than those who did not. Our finding cements on the existing evidence that in patriarchal societies, such as those found in Africa, the need to involve men right from the planning process of any reproductive health care service is critical and without them, the success of such programs might not be achieved. This finding confirms what some qualitative studies have documented regarding men’s role in influencing women’s decisions to access health services [34, 36–41], and underscores the need for innovative programs to involve men in birth preparedness and birth planning.

Our finding that women who consulted traditional birth attendants before using the motorcycle ambulances were more likely to use them highlights the crucial role that traditional birth attendants can play in influencing utilization of motorcycle ambulances on the one hand, and in increasing health facility deliveries, particularly of complicated cases, on the other. The finding also suggests that although the Ugandan Ministry of Health outlawed TBAs, their existence and operations are still valued by many pregnant women. This finding suggests a need for a re-evaluation of the importance of traditional birth attendants in convincing women with obstetric conditions to deliver at health facilities. An earlier study in Guatemala [42], and findings of a systematic review on TBA’s training for improving health behaviors and pregnancy outcomes [43] suggest that involvement of TBAs in birth preparedness reduced the incidence of post-partum complications from 7.4 to 2.5 %, thereby subsequently reducing perinatal, stillbirths and neonatal deaths. Another study found that the use of TBAs resulted in a significant increase in antenatal service utilization [44]. Collectively, these findings suggest that traditional birth attendants still have a role to play as regards maternal and child health indicators especially in developing nations such as Uganda.

This study had a number of limitations. In the first place, we only enrolled women who delivered within 2013 only, and so, our findings are restricted in time. Secondly scarcity of published literature on utilization of the motorcycle ambulances was a limitation but this was reduced by using proxy studies to identify factors that determine utilization of motorcycle ambulances by pregnant women.

## Conclusion

Our study shows that utilization of motorcycle ambulances among pregnant women is associated with older age of the mother, male involvement in the birth preparedness of a pregnant woman and consultations with traditional birth attendants. We also found that community members appreciate the use of motorcycle ambulances in referring pregnant woman. However, poor roads and cost sharing for fuel hinders their utilization. These findings suggest a need for interventions geared towards improving frequency of utilization and trust among users of the motorcycle ambulances through health education and promotion and reducing on barriers.

## Additional files

Additional file 1:
**Structured Questionnaire.** (PDF 39 kb) Additional file 2:
**Focus Group Discussion Guide.** (PDF 16 kb) Additional file 3:
**STROBE Statement.** (PDF 16 kb)

## Abbreviations

ANC: Antenatal Care
DHO: District Health Officer
FGD: Focus Group Discussion
HF: Health Facility
HMIS: Health Management Information System
LQAS: Lot Quality Assurance Survey
mA: Motorcycle Ambulances
MCH: Maternal Child Health
MOH: Ministry of Health
PONT: Partnership Overseas Networking Trust
TBRA: Traditional Birth Referral Attendants
UDHS: Uganda Demographic Health Survey
