# Supplementary material for: Factors associated with utilization of motorcycle ambulances by pregnant women in rural eastern Uganda: a cross-sectional study
Source: BMC Pregnancy Childbirth. 2016 Mar 3;16:46. doi: 10.1186/s12884-016-0808-0 (PMC4778302; doi:10.1186/s12884-016-0808-0)
Supplement: Additional file 1: — Structured Questionnaire. (PDF 39 kb) [file 12884_2016_808_MOESM1_ESM.pdf]

**Appendix I: Questionnaire for quantitative data for women who delivered in the calendar year, 2013**			
This data being collected will be used to determine the factors associated with the utilization of motorcycle ambulances by pregnant women in Mbale district. This study is being carried out by Rogers Ssebunya, a student of Makerere University, School of Public Health, offering a Masters of Public Health			
Questionnaire Number			
Date of data collection			
Names of interviewer			
**No**	**Question**	**Response**	
**Section A: socio-demographic characteristic (Please fill in or circle only once).** Qns in this section apply to **ALL** respondents.			
1	What is your age [in completed years]		
2	What is your date of birth		
3	What is your Religion	1. Anglican 2. Catholic<br>3. Muslim 4. Pentecostal<br>5. SDA 6. Others (specify)………………	
4	What is your tribe	1. Muganda<br>2. Mugisu<br>3. Ateso<br>4. Others (specify)………………………..	
5	What is your Level of education	1. No education<br>2. Primary<br>3. Secondary<br>4. More than secondary	
6	What is your occupation	1. Peasant<br>2. Student<br>3. Civil Servant<br>4. Businessman/woman<br>5. Other (specify)……………………	
7	What is your marital status	1. Never married<br>2. Married<br>3. Living together	

		4. Divorced/separated<br>5. Widowed<br>6. Other (specify)………………………….	
8	What is your average monthly Income?	1. < 50,000<br>2. 50,000 - 100,000<br>3. 100,000 – 200,000<br>4. 200,000 – 500,000<br>5. Above 500,000	
9	What does your husband occupation?	1. Peasant<br>2. Student<br>3. Civil Servant<br>4. Businessman/woman<br>5. Other (specify)……………………	
**Section B: Individual factors; Questions in this section apply to all accept Qn12, 18, 22 and 23.**			
10	In your most recent pregnancy, how did you come to the health facility to deliver?	By foot.…………………..……………..….1<br>Using a motorcycle ambulance………..……2<br>Using a boda boda……………………………3<br>Using a personal car…………………………..4<br>Using a motor vehicle ambulance…………….5<br>Other (specify)…………………………………6	
11	While you were pregnant did you know about the existence of the motorcycle ambulances?	Yes…………………………….……..1<br>No…………………………………2	If No got to 12
12	If you didn't know, How did you learn about the mAs?	1. Through the TBA<br>2. Through the neighbors<br>3. Others (specify)………………………….	
13	While you were pregnant did any member of your family have a mobile phone you could use and communicate to the motorcycle ambulance driver?	Yes..1<br>No 2	
14	During your last/current pregnancy, did/do you have a plan to deliver in a health facility	Yes..1<br>No 2	

15	Did you share your birth plan with your husband?	1. Yes<br>2. No	
16	In your last/current pregnancy, did your husband participate in decision of whether to use or not to use the motorcycle ambulance	1. Yes<br>2. No	
17	In your last/current pregnancy, how many ANC visits did you attend?	1. One<br>2. Two<br>3. Three<br>4. Four<br>5. Other [specify]…………………………	
18	If less than 4 visits, Why didn't you complete the 4 visits?	1. I didn't know<br>2. Others (specify)…………………………..	
19	History of any obstetric complication; like post partum heamorrhage, fetal distress, fetal death, post partum heamorrhage	1. Yes<br>2. No	Any of the complications mentioned means Yes
20	In your opinion, is it so important for the motorcycle ambulance to come with a midwife from the health facility once called upon by a pregnant woman in the community?	1. Strongly agree<br>2. Agree<br>3. Disagree<br>4. Strongly disagree	
21	Who would you prefer to be the driver of a motorcycle ambulance?	1. Male<br>2. Female<br>3. Doesn't matter	
22	If male or female, state reasons why you prefer the answer you have provided		
23	What motivated you to use the motorcycle ambulance?	1. My husband decision<br>2. My neighbor/friends<br>3. Easily accessible<br>4. Others (specify)………………………….	For only those who used the mA
24	Presence of a motorcycle ambulances is very essential to a pregnant woman during labour in your village	1. Strongly agree<br>2. Agree	

		3. Disagree<br>4. Strongly disagree	
25	Please give reasons why you have provided the answer in question 24 above		
26	If you were in labour pains, would you first consult a trained health care provider about using mA before coming to the health facility?	1. Yes<br>2. No	
27	If you were in labour pains, would you first consult a traditional birth attendant about using a mA before coming to the health facility?	1. Yes<br>2. No	
28	In your opinion motorcycle ambulances tend to delay to pick mothers when called upon	1. Strongly agree<br>2. Agree<br>3. Disagree<br>4. Strongly disagree	
**Section C: Community factors;** Questions in this section apply to all accept Qn 30			
29	In your culture, are women allowed to sit on motorcycles while in labour	Yes ....1<br>No 2	If No go to 30
30	If No, Would this prevent you from using the motorcycle ambulances?	Yes………………………………1<br>No……………………………….2	
31	Would it bother you if community members saw you being transported on a motorcycle ambulance while in labour?	1. Yes<br>2. No	
**Section D: Health service factors;** Questions in this section apply to ALL accept 39, 40 and 41			
32	In your last pregnancy did you find drugs/supplies you need for delivery?	Yes…………………………...…1<br>No ……………………..….…2	
33	In last pregnancy, did you discuss the decision on how to come to the health facility to deliver with the TBA?	1. Yes<br>2. No	
34	In your opinion motorcycle ambulance drivers' attitudes towards women are very poor	1. Strongly agree<br>2. Agree<br>3. Disagree	

		4. Strongly disagree	
35	Is the health facility with motorcycle ambulance is very far away from you home	1. Strongly agree<br>2. Agree<br>3. Disagree<br>4. Strongly disagree	
36	Health workers at health facilities with motorcycle ambulances are not always available	1. Strongly agree<br>2. Agree<br>3. Disagree<br>4. Strongly disagree	
37	In your last pregnancy, health workers attitudes towards pregnant women were not good	1. Strongly agree<br>2. Agree<br>3. Disagree<br>4. Strongly disagree	
38	In your opinion, mA drivers have obstetric skills to help a woman in case she is in labour while in transit	No……..………………………..…1<br>Yes ………………………..……2<br>I don't know…..………………...3	
39	What are some of the reasons why you never used a motorcycle ambulance when you were in labour?	1. My husband declined<br>2. We had our personal means of transport<br>3. mA driver declined to come<br>4. I was asked to pay for fuel<br>5. Other (specify)………………………..	For only those who didn't use the mA
40	If motorcycle ambulance was the only means of transport in your village, would you use it to come for delivery?	Yes…………………………...…1<br>No ………………………..……2	For only those who didn't use the mA
41	If No, why wouldn't you use it?	1. It is not comfortable<br>2. It is very slow<br>3. It takes a long time to reach when called<br>4. Others (specify)………………………	For only those who didn't use the mA
