# Supplementary material for: Factors associated with utilization of motorcycle ambulances by pregnant women in rural eastern Uganda: a cross-sectional study
Source: BMC Pregnancy Childbirth. 2016 Mar 3;16:46. doi: 10.1186/s12884-016-0808-0 (PMC4778302; doi:10.1186/s12884-016-0808-0)
Supplement: Additional file 2: — Focus Group Discussion Guide. (PDF 16 kb) [file 12884_2016_808_MOESM2_ESM.pdf]

Appendix II: Focus group discussion guide:

Good morning Sir, Madam

I am called Rogers Ssebunya, a student from Makerere University school of Public health. I am conducting a study about the factors associated with the utilization of motorcycle ambulances by pregnant women in Mbale district. I am requesting you to participate in a focus group discussion as you are one of the community members served by a health facility with a motorcycle ambulance.

Findings from this study will assist the health workers where the ambulances are located, DHT or Mbale, MOH and the community as a whole. Results from this discussion will strictly be for academic purposed and will remain confidential. Recordings made during the discussions will be destroyed after transcription is done. You have the right to refuse or withdraw from the study anytime you feel like.

Questions:

Knowledge about the use of motorcycle ambulances

1. How many of you have heard about the motorcycle ambulances being used to refer pregnant women to health facilities?
2. What do you know about these ambulances?
3. Talk about how these ambulances work within your communities
4. What kind of pregnant women use the motorcycle ambulances?

Opinions and attitudes towards motorcycle ambulances

5. What is your opinion on the use of motorcycle ambulances in referring pregnant women to health facilities?
6. If you were pregnant this year, would you use these ambulances to go to the health center?
7. In your neighborhood, what do people comment about the motorcycle ambulances?

Facilitators and Barriers for using motorcycle ambulances

8. What could motivate you to use the motorcycle ambulances?
9. What don't you like about these ambulances?

Suggestions and recommendations about the motorcycle ambulances

10. In your opinion, what would you change on the motorcycle ambulances or the way they are being used?
11. In which ways do you think we can improve the use the motorcycle ambulance in our communities?
