# Supplementary material for: Factors associated with utilization of motorcycle ambulances by pregnant women in rural eastern Uganda: a cross-sectional study
Source: BMC Pregnancy Childbirth. 2016 Mar 3;16:46. doi: 10.1186/s12884-016-0808-0 (PMC4778302; doi:10.1186/s12884-016-0808-0)
Supplement: Additional file 3: — STROBE Statement. (PDF 16 kb) [file 12884_2016_808_MOESM3_ESM.pdf]

STROBE Statement – Study REF: MS: 1416884362157330

	Item No	Recommendation	
**Title and abstract**	1	(*a*) Indicate the study's design with a commonly used term in the title or the abstract	This was included in the title (page 1 line 2) and in the abstract (page 2 line 8)
		(*b*) Provide in the abstract an informative and balanced summary of what was done and what was found	This summary was provided on pages 2-3
**Introduction**			
Background/rationale	2	Explain the scientific background and rationale for the investigation being reported	Background and rationale of doing this study are embedded in the segments from page 5 line 11 to page 6 line 5.
Objectives	3	State specific objectives, including any prespecified hypotheses	The main objective of this study is documented on page 6 line 3-5
**Methods**			
Study design	4	Present key elements of study design early in the paper	Details of these were provided on page 6 line 9-18
Setting	5	Describe the setting, locations, and relevant dates, including periods of recruitment, exposure, follow-up, and data collection	Study setting and locations are included on page 6 line 10-18. Information on data collection period is included on page 9 line 12.
Participants	6	(*a*) Give the eligibility criteria, and the sources and methods of selection of participants	Eligibility criterion for the quantitative objective is indicated on page 8 lines 12-15 while for the qualitative objective it is on page 8 line 23 and page 9 lines 1-3.
Variables	7	Clearly define all outcomes, exposures, predictors, potential confounders, and effect modifiers. Give diagnostic criteria, if applicable	In this study, the outcome variable was "*used or didn't a motorcycle ambulance*". This has been included on page 10 lines 5-7. Predictors were included on page 9 lines 14 – 23 and on page 10 lines 1-5.
Data sources/ measurement	8*	For each variable of interest, give sources of data and details of methods of assessment (measurement). Describe comparability of assessment methods if there is more than one group	Details of women to be interviewed were indicated on page 8 line 9. Details of measurements of the predictors were provided on page 11 lines 9-20
Bias	9	Describe any efforts to address potential sources of bias	In this study we minimised recall bias by selecting women who had given birth in the previous calendar year. We also further minimised selection bias by randomly selecting the women to interview using a table of random numbers. These efforts are on page 8 line 11-18

Study size	10	Explain how the study size was arrived at	Assumptions used in estimating the sample size have were included on page 8 lines 5-9
Quantitative variables	11	Explain how quantitative variables were handled in the analyses. If applicable, describe which groupings were chosen and why	Details of how the outcome and independent variables were handled were provided on page 12 lines 1-11.
Statistical methods	12	(*a*) Describe all statistical methods, including those used to control for confounding	Details of controlling for confounding were provided for on page 12 line 7.
		(*b*) Describe any methods used to examine subgroups and interactions	Not applicable
		(*c*) Explain how missing data were addressed	Missing data was minimised right from the data collection period.
		(*d*) If applicable, describe analytical methods taking account of sampling strategy	Not applicable
		(*e*) Describe any sensitivity analyses	We didn't carry out any sensitivity analysis
**Results**			
Participants	13*	(a) Report numbers of individuals at each stage of study—eg numbers potentially eligible, examined for eligibility, confirmed eligible, included in the study, completing follow-up, and analysed	In this study eligibility was at one stage. Details of eligibility are on page 8 lines 12-15
		(b) Give reasons for non-participation at each stage	Not applicable
		(c) Consider use of a flow diagram	Not applicable
Descriptive data	14*	(a) Give characteristics of study participants (eg demographic, clinical, social) and information on exposures and potential confounders	Demographic characteristics were provided under socio demographic characteristics on page 13 line 8-16. Potential confounders included religion, tribe and average monthly income.
		(b) Indicate number of participants with missing data for each variable of interest	We had no participant with incomplete data in this study. This was sorted out at data collection time where all missing data was obtained.
Outcome data	15*	Report numbers of outcome events or summary measures	We only one outcome measure in this study – use or didn't use a motorcycle ambulance
Main results	16	(*a*) Give unadjusted estimates and, if applicable, confounder-adjusted estimates and their precision (eg, 95% confidence interval). Make clear which confounders were adjusted for and why they were included	Unadjusted and adjusted estimates were provided in table 4 page 30. Distance and ANC visits were some of the adjusted for confounders.

		(*b*) Report category boundaries when continuous variables were categorized	In this study age was categorised and details were shown in tables 1-4.
		(*c*) If relevant, consider translating estimates of relative risk into absolute risk for a meaningful time period	Our measures of association was OR. We didn't have any relative risk measure of association.
Other analyses	17	Report other analyses done—eg analyses of subgroups and interactions, and sensitivity analyses	All the analyses that were done, findings were reported in the tables 1-4.
**Discussion**			
Key results	18	Summarise key results with reference to study objectives	Summary of key results were done per each objective in the results section starting from page 13.
Limitations	19	Discuss limitations of the study, taking into account sources of potential bias or imprecision. Discuss both direction and magnitude of any potential bias	Limitations of the study were included on page 20 lines 4-8.
Interpretation	20	Give a cautious overall interpretation of results considering objectives, limitations, multiplicity of analyses, results from similar studies, and other relevant evidence	An overall interpretation was included in the conclusion on page 20 line 11-18
Generalisability	21	Discuss the generalisability (external validity) of the study results	Our findings target users of motorcycle ambulance who are mainly women. Hence they can be generalised. This is shown on page 20 lines 17-18.
**Other information**			
Funding	22	Give the source of funding and the role of the funders for the present study and, if applicable, for the original study on which the present article is based	This was a study purely funded by the investigator. There was no external funding.

*Give information separately for exposed and unexposed groups.
